# Supplementary material for: Somatically hypermutated antibodies isolated from SARS-CoV-2 Delta infected patients cross-neutralize heterologous variants
Source: Nat Commun. 2023 Feb 24;14:1058. doi: 10.1038/s41467-023-36761-0 (PMC9951844; doi:10.1038/s41467-023-36761-0)
Supplement: Supplementary file 1 — Supplementary Information [file 41467_2023_36761_MOESM1_ESM.pdf]

Supplementary Information

Somatically hypermutated antibodies isolated from SARS-CoV-2 Delta infected patients cross-neutralize heterologous variants

Yu et al.

Supplementary Information includes:

Supplementary Figures 1-12
Supplementary Tables 1-6
Supplementary References

Supplementary Figures

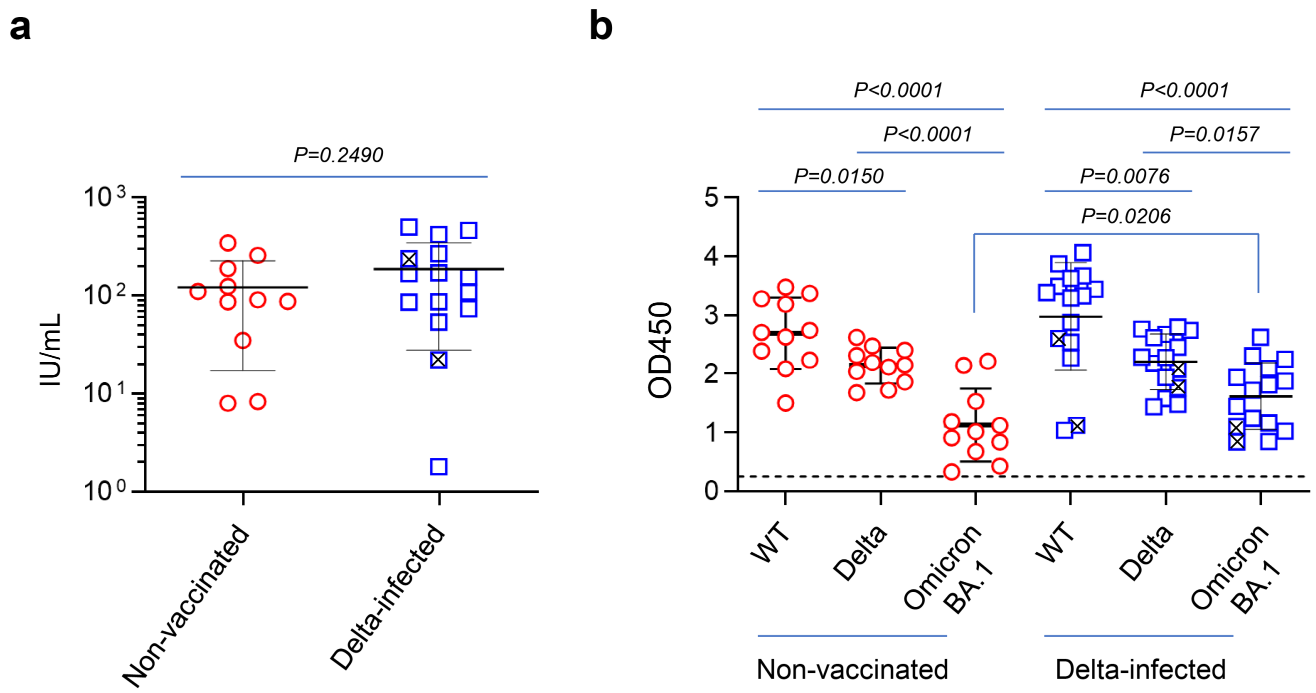

Supplementary Fig. 1 | Reactivity of SARS-CoV-2 convalescent plasma to three SARS-CoV-2 RBD antigens. a. SARS-CoV-2 convalescent plasma antibody titers were measured using a chemiluminescence kit against wild type (WT) RBD. Samples information and antibody titers are summarized in **Supplementary Table 1**. **b**. RBD binding by SARS-CoV-2 convalescent plasmas was measured by ELISA against three SARS-CoV-2 RBDs. Plasma binding activities against WT, Delta (B.1.617.2) and Omicron BA.1 (B.1.1.529) are represented as OD450 values from ELISA. Non-vaccinated, n = 11 patients; Delta-infected, n = 15 patients. P values are calculated by two-tailed unpaired Student's t-test. Data are presented as mean values ± SD. Data for Delta-infected donors without vaccine are marked with "×" and not used for statistics. Source data for **b** are provided as a Source Data file.

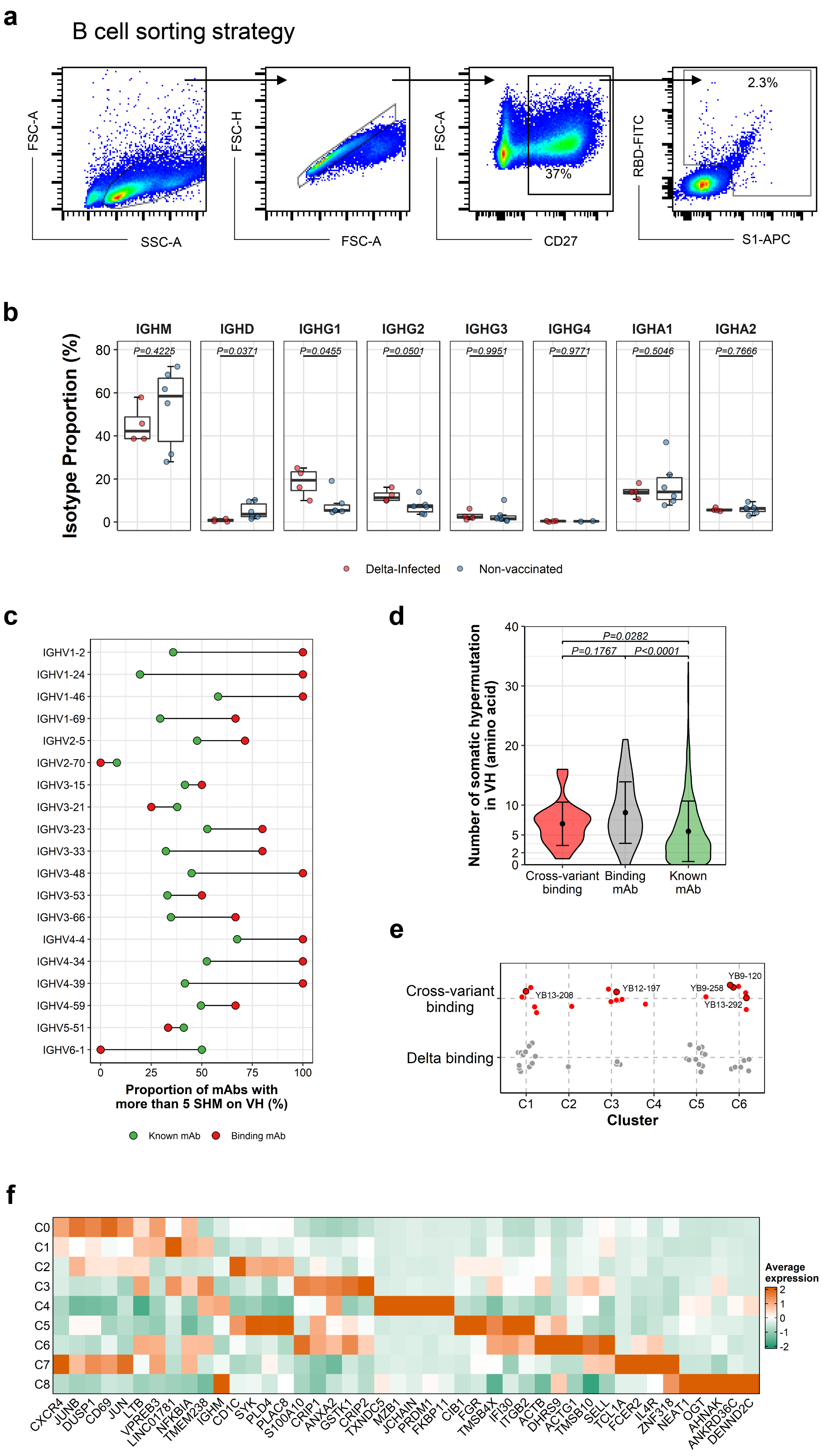
a
B cell sorting strategy
FSC-A
SSC-A
FSC-H
FSC-A
FSC-A
CD27
37%
RBD-FITC
S1-APC
2.3%
b
Isotype Proportion (%)
IGHM
IGHD
IGHG1
IGHG2
IGHG3
IGHG4
IGHA1
IGHA2
P=0.4225
P=0.0371
P=0.0455
P=0.0501
P=0.9951
P=0.9771
P=0.5046
P=0.7666
Delta-Infected
Non-vaccinated
c
IGHV1-2
IGHV1-24
IGHV1-46
IGHV1-69
IGHV2-5
IGHV2-70
IGHV3-15
IGHV3-21
IGHV3-23
IGHV3-33
IGHV3-48
IGHV3-53
IGHV3-66
IGHV4-4
IGHV4-34
IGHV4-39
IGHV4-59
IGHV5-51
IGHV6-1
Proportion of mAbs with more than 5 SHM on VH (%)
Known mAb
Binding mAb
d
Number of somatic hypermutation in VH (amino acid)
P=0.0282
P=0.1767
P<0.0001
Cross-variant binding
Binding mAb
Known mAb
e
Cross-variant binding
Delta binding
YB13-208
YB12-197
YB9-258
YB9-120
YB13-292
C1
C2
C3
C4
C5
C6
Cluster
f
C0
C1
C2
C3
C4
C5
C6
C7
C8
Average expression
CXCR4
JUNB
DUSP1
CD69
JUN
LTB
VPREB3
LINC01781
NFKBIA
TMEM238
IGHM
CD1C
SYK
PLD4
PLAC8
S100A10
CRIP1
ANXA2
GSTK1
CRIP2
TXNDC5
MZB1
JCHAIN
PRDM1
FKBP11
CIB1
FGR
TMSB4X
IFI30
ITGB2
ACTB
DHRS9
ACTG1
TMSB10
SELL
TCL1A
FCER2
IL4R
ZNF318
NEAT1
OGT
AHNAK
ANKRD36C
DENND2C

Supplementary Fig. 2 | B cell isolation, mAb distributions and heatmap of marker genes. **a**, Gating strategy for isolation of memory B cells binding to SARS-CoV-2 Delta variant S1 protein ($S1^+$) and RBD subunit (RBD^+) from PBMCs of COVID-19 convalescent patients. Enriched $CD19^+$ B cells were first gated by FSC-A and SSC-A and doublets were excluded by the FSC-H and FSC-A. The RBD^+ $S1^+$ B cells were isolated from $CD27^+$ memory B cells. **b**, Proportion of 8 isotypes in each pooled sample (Delta-Infected, n = 4 pooled samples; Non vaccinated, n = 6 pooled samples), related to **Fig. 1e**. P values are calculated by two-tailed unpaired Student's t-test. The boxplots depict the median (horizontal line), upper/lower quartiles (boxes), and range (whiskers). **c**, Proportion of mAbs with more than 5 SHM on VH sequences (mean value of known mAbs) for each V gene family, compared between Delta-binding mAbs and known mAbs. **d**, Violin plot showing SHM counts on VH sequences of 22 crossing-variant binding antibodies, 63 binding antibodies and known mAbs. P values are calculated by two-sided Mann-Whitney U test. Data are presented as mean values ± SD. **e**, Distribution of cross-reactive mAbs (red dot) and binding mAbs (grey dot) in each cluster. Five most potent cross-neutralizing mAbs are highlighted (black circle). **f**, Mean expression levels of top marker genes for each cluster as shown in **Fig. 1b**. Source data for **b**, **c** and **f** are provided as a Source Data file.

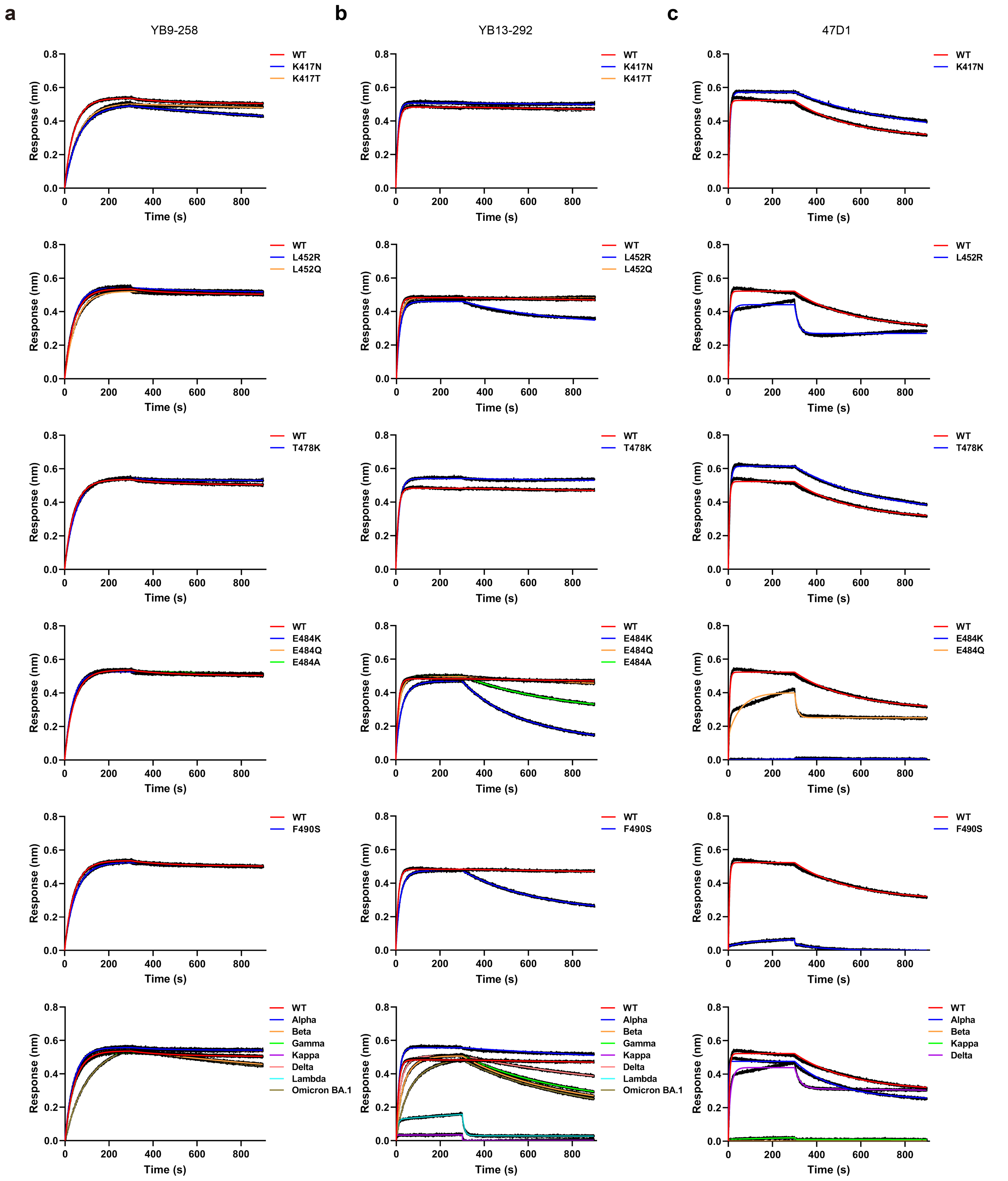

Supplementary Fig. 3 | Effect of common RBD mutations on binding of YB9-258, YB13-292 and 47D1. a, YB9-258, **b**, YB13-292, **c**, 47D1 were immobilized onto Protein A biosensors. Wildtype RBD and RBDs with indicated mutations were diluted to 200 nM. Sensors immobilized with IgGs were submerged into RBD solutions to record antigen association and dissociation. Association and dissociation rates (k_{on}, k_{off}) were calculated by curve fitting, dissociation constants (K_D) were calculated as k_{off}/k_{on}, these kinetic parameters are present in

Supplementary Table 3. Fold changes in dissociation constants (K_D) affected by different RBD mutations for YB9-258 and YB13-292 are shown in **Fig. 2c**. These are calculated by normalization to K_Ds calculated from binding reactions of YB9-258 and YB13-292 to wildtype RBD.

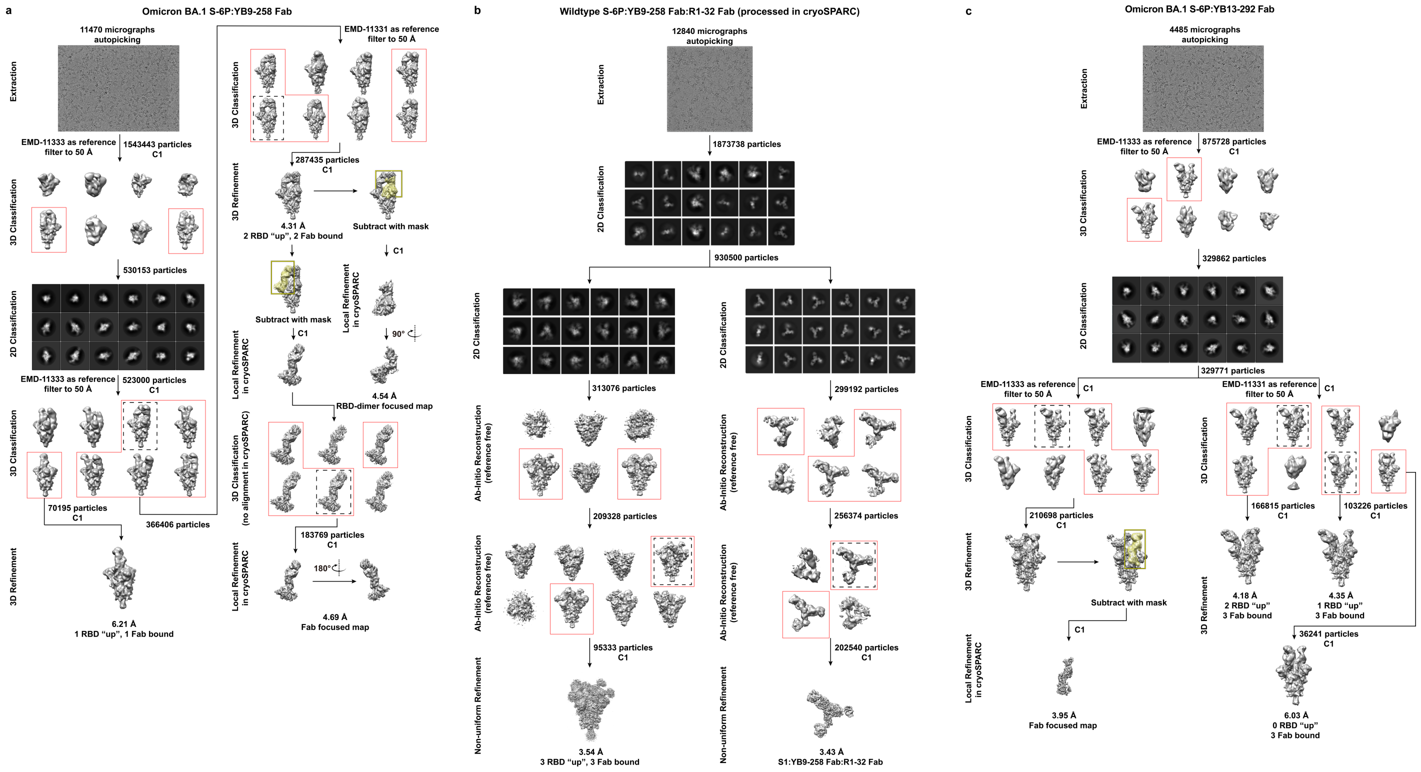

Supplementary Fig. 4 | Cryo-EM data processing pipelines. a, Data processing pipeline for the Omicron BA.1 S-6P:YB9-258 Fab dataset. **b**, Data processing pipeline for the Wildtype S-6P:YB9-258 Fab:R1-32 Fab dataset. **c**, Data processing pipeline for the Omicron BA.1 S-6P:YB13-292 Fab dataset. Red boxes indicate selected classes. Dashed boxes indicate structures used as references for classification/refinement. Subtraction masks are colored in yellow.

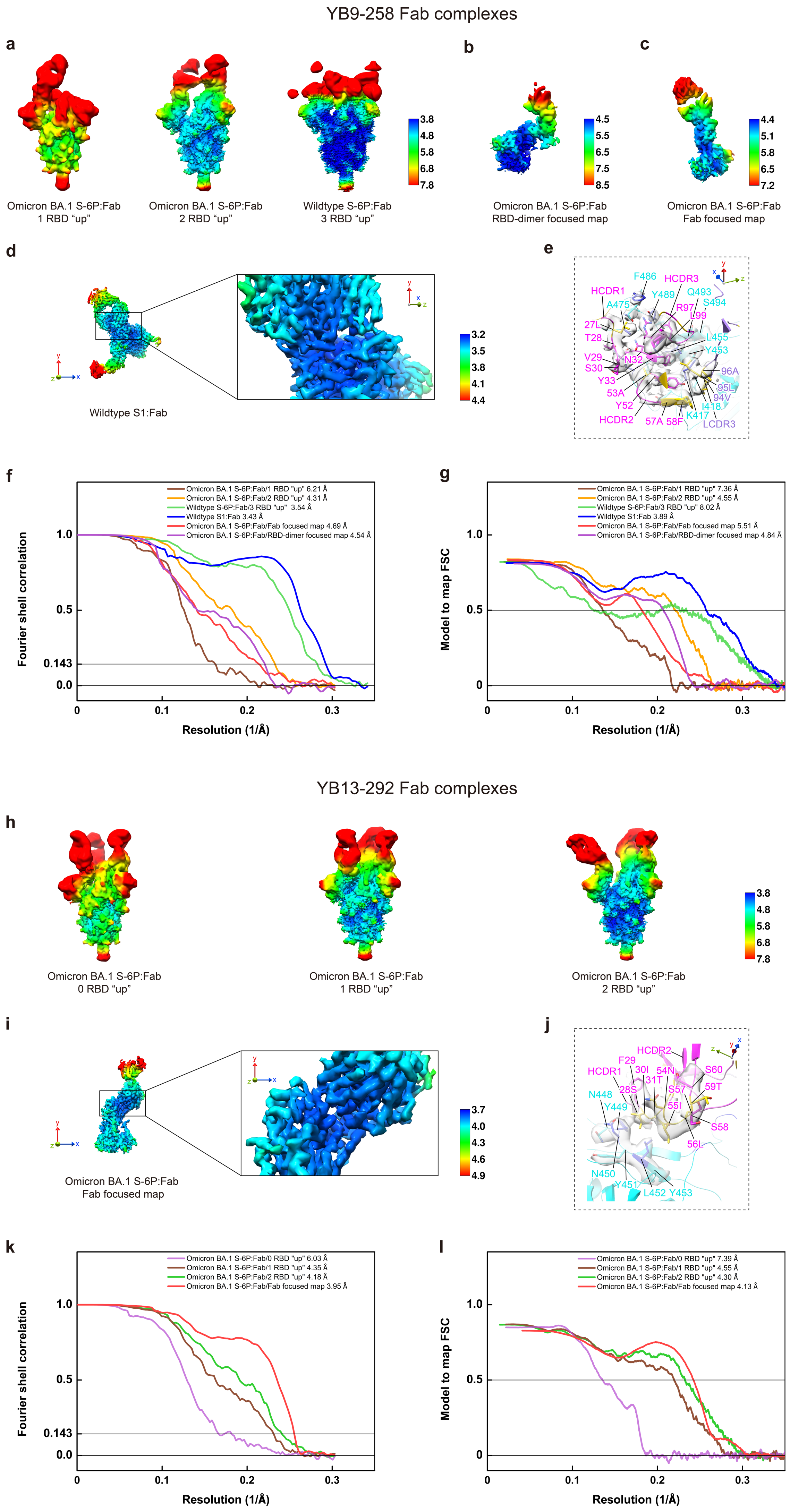

YB9-258 Fab complexes
a
Omicron BA.1 S-6P:Fab 1 RBD "up"
Omicron BA.1 S-6P:Fab 2 RBD "up"
Wildtype S-6P:Fab 3 RBD "up"
3.8 4.8 5.8 6.8 7.8
b
Omicron BA.1 S-6P:Fab RBD-dimer focused map
4.5 5.5 6.5 7.5 8.5
c
Omicron BA.1 S-6P:Fab Fab focused map
4.4 5.1 5.8 6.5 7.2
d
Wildtype S1:Fab
3.2 3.5 3.8 4.1 4.4
e
F486 HCDR3 HCDR1 A475 Y489 Q493 S494 R97 L99 27L T28 L455 Y453 V29 N32 S30 96A Y33 95L 53A 94V Y52 I418 K417 HCDR2 57A 58F LCDR3
f
Omicron BA.1 S-6P:Fab/1 RBD "up" 6.21 Å
Omicron BA.1 S-6P:Fab/2 RBD "up" 4.31 Å
Wildtype S-6P:Fab/3 RBD "up" 3.54 Å
Wildtype S1:Fab 3.43 Å
Omicron BA.1 S-6P:Fab/Fab focused map 4.69 Å
Omicron BA.1 S-6P:Fab/RBD-dimer focused map 4.54 Å
Fourier shell correlation
Resolution (1/Å)
g
Omicron BA.1 S-6P:Fab/1 RBD "up" 7.36 Å
Omicron BA.1 S-6P:Fab/2 RBD "up" 4.55 Å
Wildtype S-6P:Fab/3 RBD "up" 8.02 Å
Wildtype S1:Fab 3.89 Å
Omicron BA.1 S-6P:Fab/Fab focused map 5.51 Å
Omicron BA.1 S-6P:Fab/RBD-dimer focused map 4.84 Å
Model to map FSC
Resolution (1/Å)
YB13-292 Fab complexes
h
Omicron BA.1 S-6P:Fab 0 RBD "up"
Omicron BA.1 S-6P:Fab 1 RBD "up"
Omicron BA.1 S-6P:Fab 2 RBD "up"
3.8 4.8 5.8 6.8 7.8
i
Omicron BA.1 S-6P:Fab Fab focused map
3.7 4.0 4.3 4.6 4.9
j
HCDR2 F29 HCDR1 30I 54N S60 31T 28S S57 59T N448 Y449 55I S58 56L N450 Y451 L452 Y453
k
Omicron BA.1 S-6P:Fab/0 RBD "up" 6.03 Å
Omicron BA.1 S-6P:Fab/1 RBD "up" 4.35 Å
Omicron BA.1 S-6P:Fab/2 RBD "up" 4.18 Å
Omicron BA.1 S-6P:Fab/Fab focused map 3.95 Å
Fourier shell correlation
Resolution (1/Å)
l
Omicron BA.1 S-6P:Fab/0 RBD "up" 7.39 Å
Omicron BA.1 S-6P:Fab/1 RBD "up" 4.55 Å
Omicron BA.1 S-6P:Fab/2 RBD "up" 4.30 Å
Omicron BA.1 S-6P:Fab/Fab focused map 4.13 Å
Model to map FSC
Resolution (1/Å)

Supplementary Fig. 5 | Resolution assessment of cryo-EM structures. a-d, Density maps of YB9-258 complexes showing local resolutions. **e**, Representative cryo-EM density around the YB9-258 epitope in the Wildtype S1:YB9-258 complex map (**d**). **h-i**, Density maps of YB13-292 complexes showing local resolutions. **j**, Representative cryo-EM density around the YB13-292 epitope in the Omicron BA.1 S-6P:YB13-292 complex focused map (**i**). **f and k**, Global resolution assessment by Fourier shell correlation at the 0.143 criterion. **g and l**, Correlations of model vs map by Fourier shell correlation (FSC) at the 0.5 criterion.

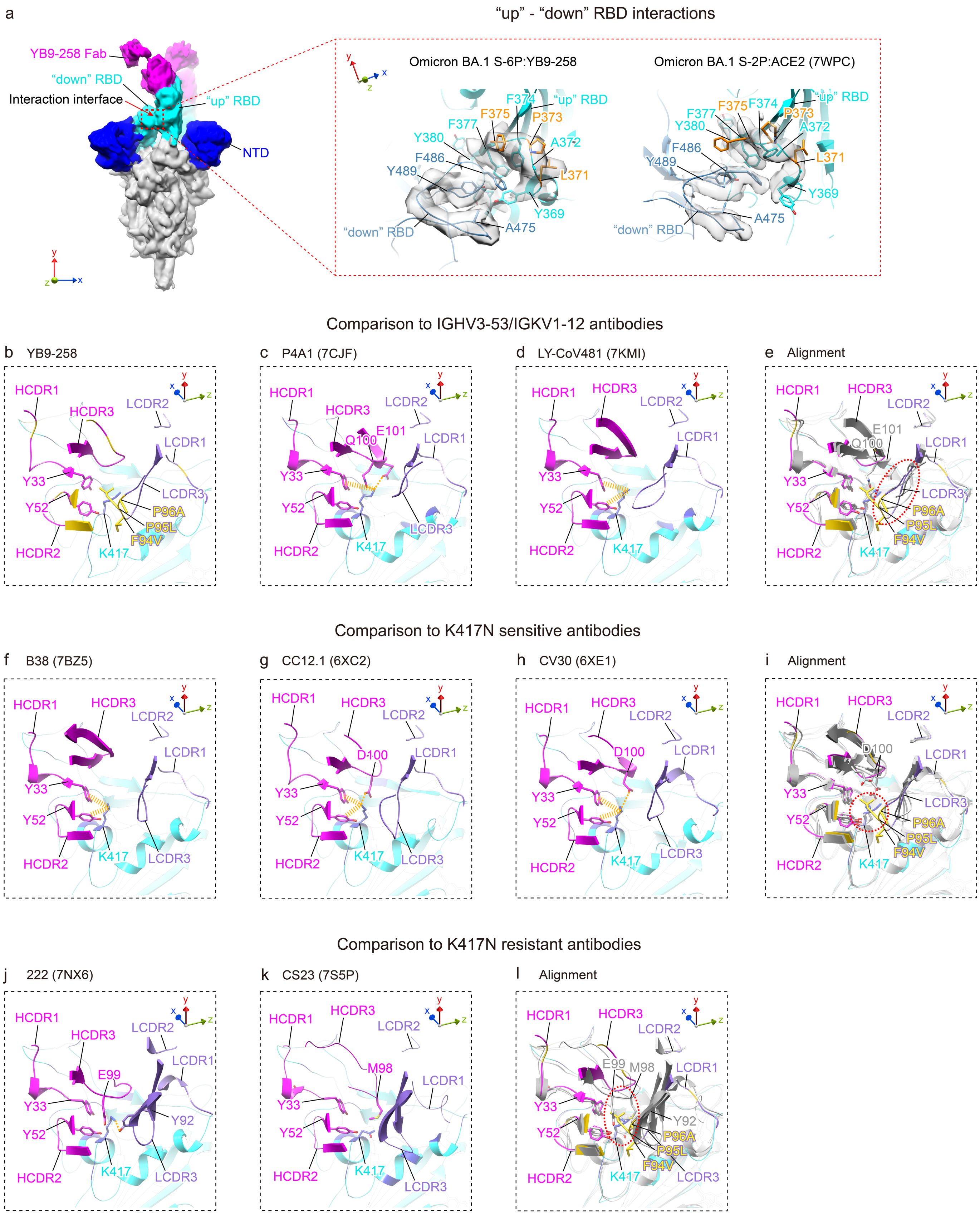

Supplementary Fig. 6 | Structural features of YB9-258:spike complexes and comparisons to other VH3-53 antibodies. a, Interactions between "up"-"down" RBDs in the Omicron BA.1 S-6P:YB9-258 2 RBD "up" structure. Left panel, the interaction site (indicated by the red box) between "up"-"down" RBDs in the overall structure of Omicron BA.1 S-6P:YB9-258 2 RBD "up" complex. Middle panel, a zoomed in view showing the Omicron BA.1 specific S371L, S373P, and S375F substitutions exposed hydrophobic surface in "up" RBD interacting with

residues in “down” RBD, two RBD are shown different shades of cyan, transparent surfaces show cryo-EM density of RBD-dimer focused map (**Supplementary Fig. 5b**). Right panel, previously observed interactions between “up”-“down” RBDs in Omicron BA.1 S-2P:ACE2 complex structures (PDB: 7WPC, https://www.rcsb.org/structure/7WPC)[1], which were proposed to affect receptor binding. **b-d**, Structures of epitopes and their interactions with structurally characterized IGH3-53/IGKV1-12 antibodies. Cation-π interactions and salt bridges are indicated by thick and thin dashed lines respectively. **e**, Overlap of IGH3-53/IGKV1-12 antibody structures. YB9-258 structure is colored, P4A1 and LY-CoV481 structures are shown in grey. The overlap revealed that LCDR3 of YB9-258 adopts a different conformation engaging a closer contact to RBD (highlighted in the dashed red oval). **f-h**, Structures of epitopes and their interactions with structurally characterized VH3-53 antibodies known to be escaped by the K417N RBD substitution. **i**, Overlap of YB9-258 and K417N sensitive antibody structures. YB9-258 structure is colored, B38, CC12.1, and CV30 structures are shown in grey. The sidechain of K417 in the YB9-258:Wildtype S-6P structure adopts a different orientation rendering itself unable to form interaction to Y33 and Y52 (highlighted in the dashed red circle). **j-k**, Structures of epitopes and their interactions with structurally characterized VH3-53 antibodies known to resist the K417N RBD substitution. **l**, Overlap of YB9-258 and known K417N resistant antibody structures. YB9-258 structure is colored, 222 and CS23 structures are shown in grey. Residues can hinder K417 from interacting with Y33 and Y52, including P95L in YB9-258 LCDR3, E99 in 222 HCDR3 and M98 in CS23 HCDR3 are shown (highlighted in the dashed red oval).

YB9-258 heavy chain

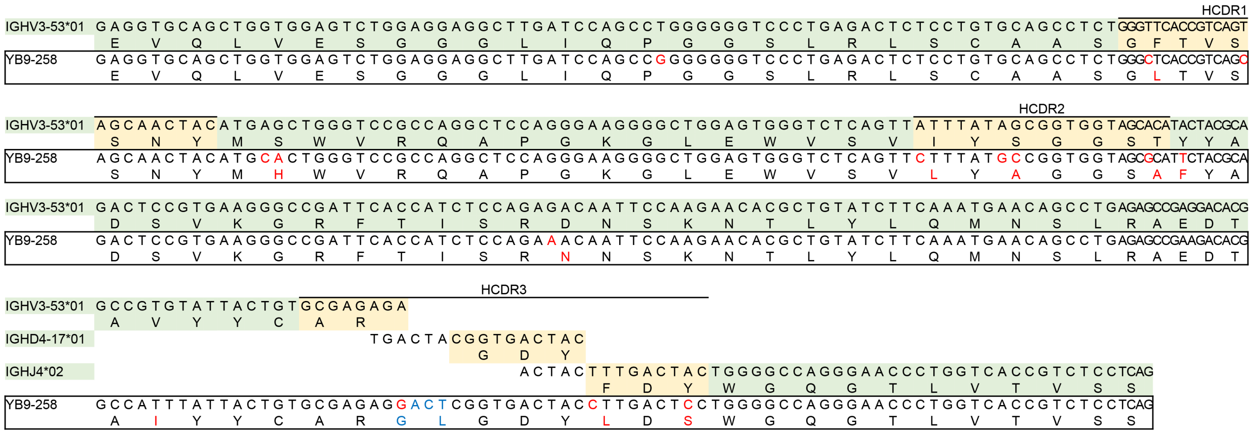

YB9-258 light chain

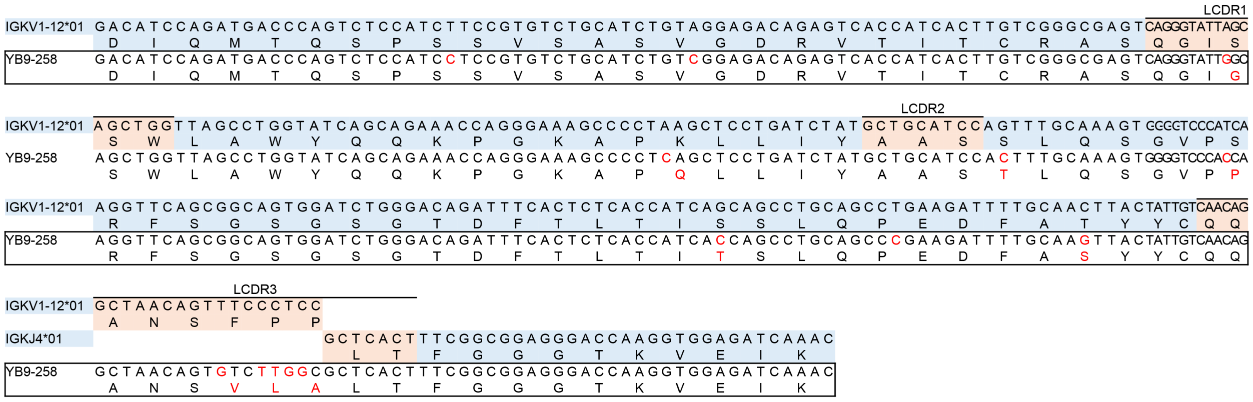

YB13-292 heavy chain

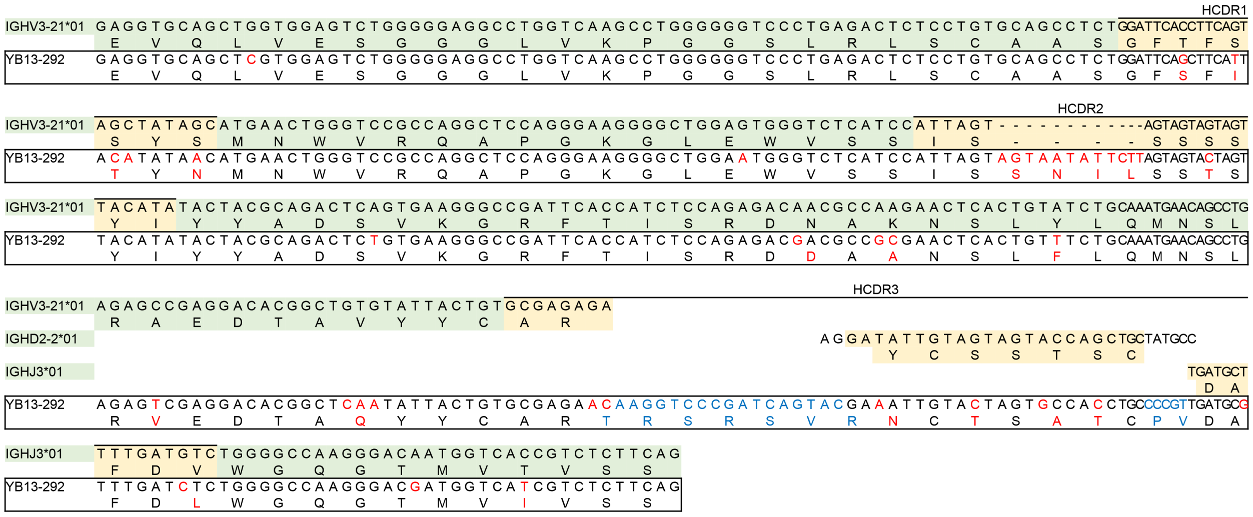

YB13-292 light chain

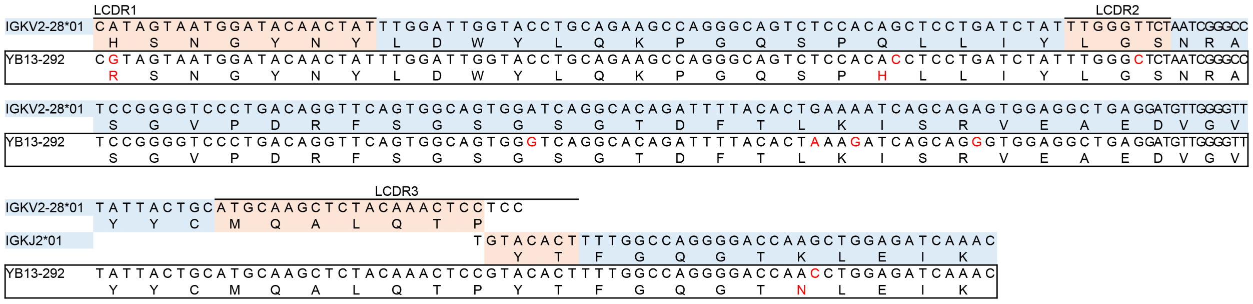

Supplementary Fig. 7 | Sequences of YB9-258 and YB13-292 compared to their respective germline sequences. For YB9-258 antibody, heavy chain sequence is compared to IGHV3-53*01, IGHD4-17*01 and IGHJ4*02 germline genes. Light chain sequence is compared to IGKV1-12*01, IGKJ4*01 germline genes. For YB13-292 antibody, heavy chain sequence is compared to IGHV3-21*01, IGHD2-2*01 and IGHJ3*01 germline genes. Light chain sequence is compared to IGKV2-28*01, IGKJ2*01 germline genes. Putative nucleotides and amino acids generated through somatic hypermutations are colored red. Nucleotide sequences predicted to have derived from VDJ recombination and their encoded amino acids are colored blue.

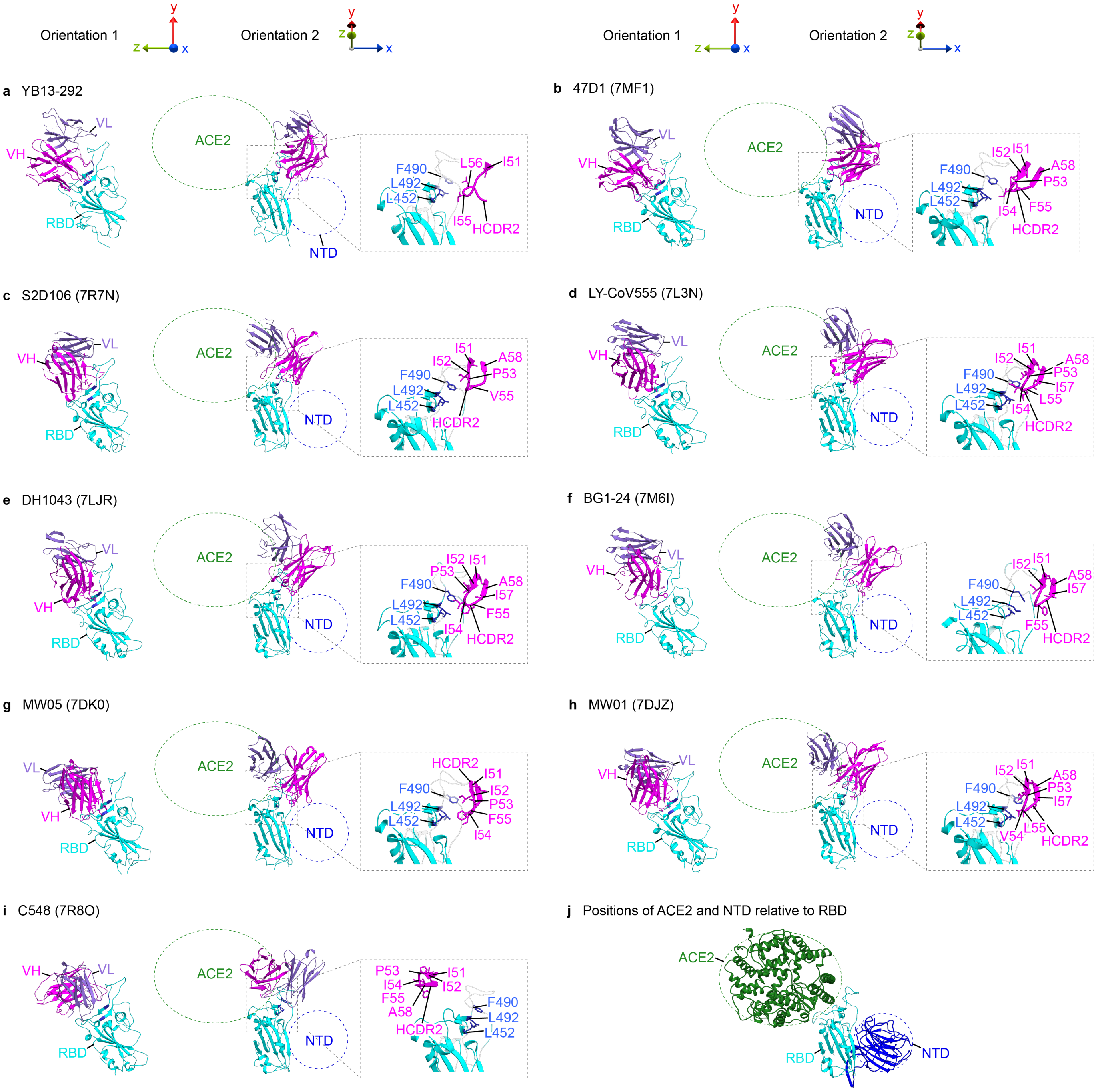

Supplementary Fig. 8 | Binding mode of YB13-292 compared to other "class 2" VH1-69 antibodies. a-i, Fab-VH, Fab-VL and RBD are colored in magenta, purple, and cyan, respectively. The green circles show the position of ACE2 when bound to RBD; the blue circles show the position of NTD in relation to an RBD in a "down" position as illustrated in panel **j**. Epitopes of HCDR2 and hydrophobic residues involved in YB13-292 and selected VH1-69[2] antibody-antigen interaction are shown in dashed boxes. Except for **i**, C548, all shown antibodies interacting with L452, F490, and L492 using their hydrophobic HCDR2.

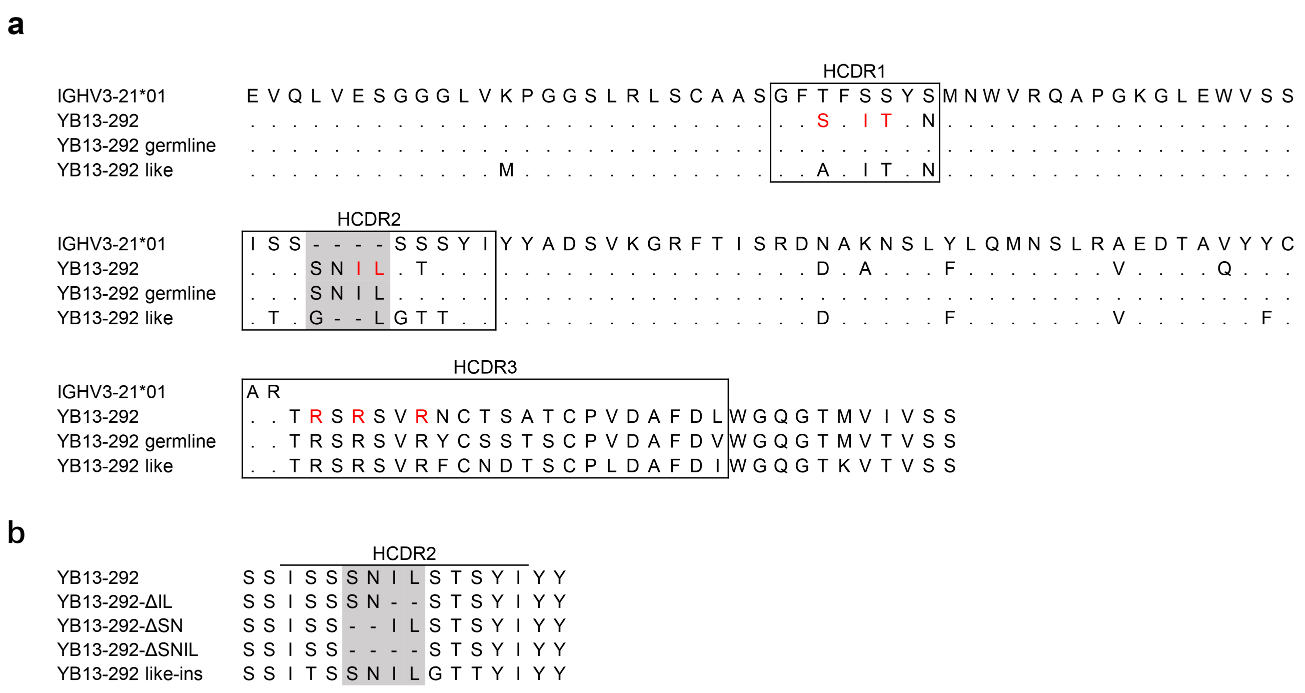

Supplementary Fig. 9 | HCDR2 insertion in YB13-292 and antibody sequences related to YB13-292. a, Sequence alignment of the IGHV3-21*01 gene against heavy chains of YB13-292, YB13-292 germline precursor, and an antibody highly similar to YB13-292 (YB13-292 like). YB13-292 germline precursor is generated by reverting all point substitutions according to the inferred germline gene and the YB13-292 like antibody sequence was identified from the single-cell BCR repertoire by similarity as described in the Methods section. HCDR2 insertions are shaded in dark grey. CDR loops defined according to IMGT definition is boxed. Dots indicate identical amino acids and dashes indicate gaps. The identified paratope residues mapped onto the heavy chain sequence of YB13-292 were colored in red. **b**, Sequence alignment of HCDR2 region of YB13-292 showing different variations of the "SNIL" insertion engineered in this study (YB13-292-ΔIL, YB13-292-ΔSN), or YB13-292 without the insertion (YB13-292-ΔSNIL) and the YB13-292 like antibody containing the "SNIL" insertion (YB13-292 like-ins).

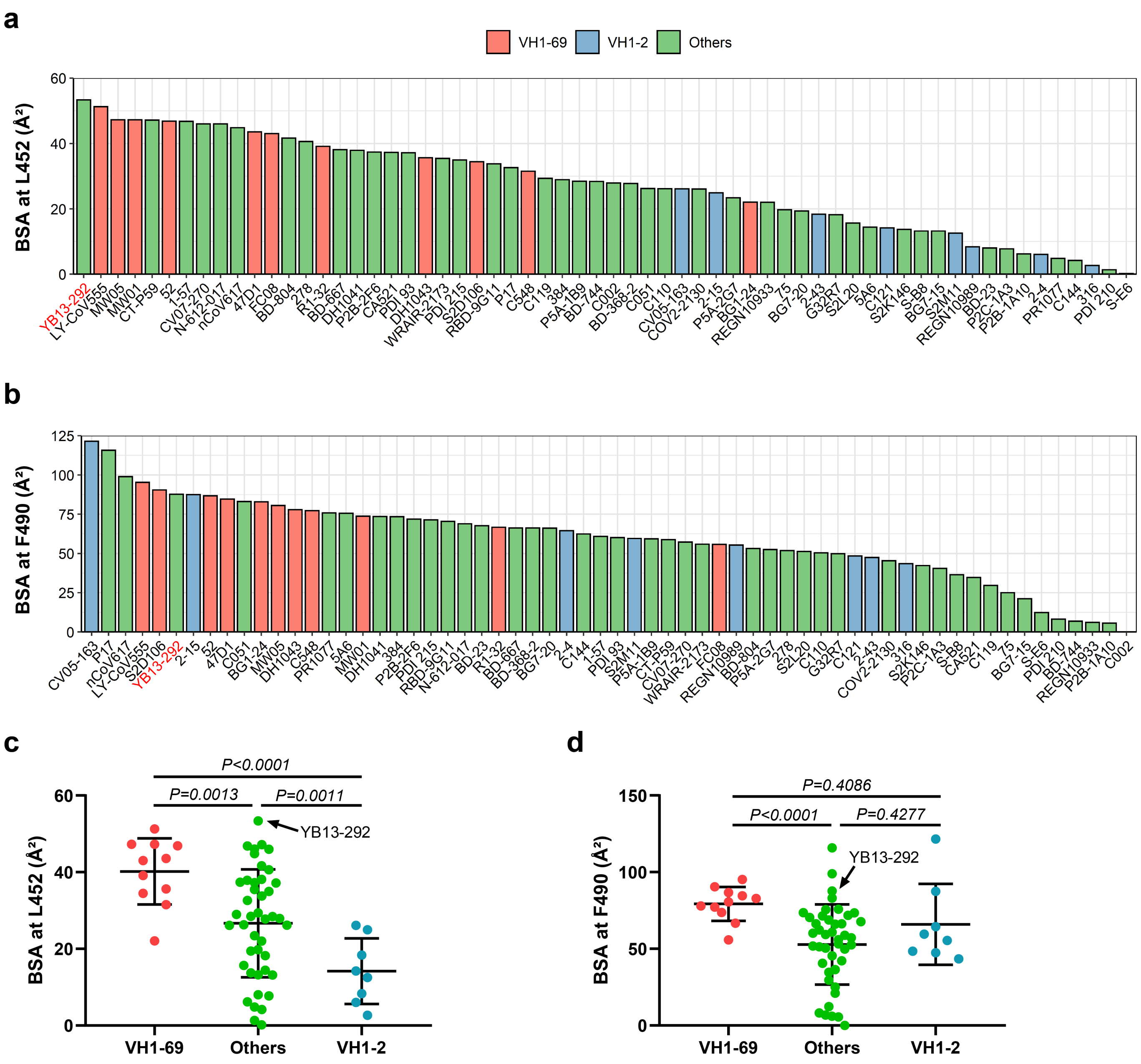

Supplementary Fig. 10 | Analysis of 452, 490 binding among currently structurally characterized 452 contacting antibodies. Structures of SARS-CoV-2 RBD targeting antibodies contacting residue 452 were identified from the PDB. Buried surface areas (BSA) at L452 were calculated using PISA[3]. **a**, Histogram showing the buried surface area at L452 by L452 contacting mAbs. **b**, Histogram showing the buried surface area at F490 by L452 contacting mAbs. **c-d**, Comparison of buried surface areas at L452 and F490 among different antibody groups (VH1-69, n = 11; Others, n = 43; VH1-2, n = 8), VH1-2 antibodies known to preferentially encode "class 2" antibodies are used as a comparison group. P values are calculated by one-way ANOVA with Game Howell's multiple comparisons test. Data are presented as mean values ± SD. Source data for **c** and **d** are provided as a Source Data file.

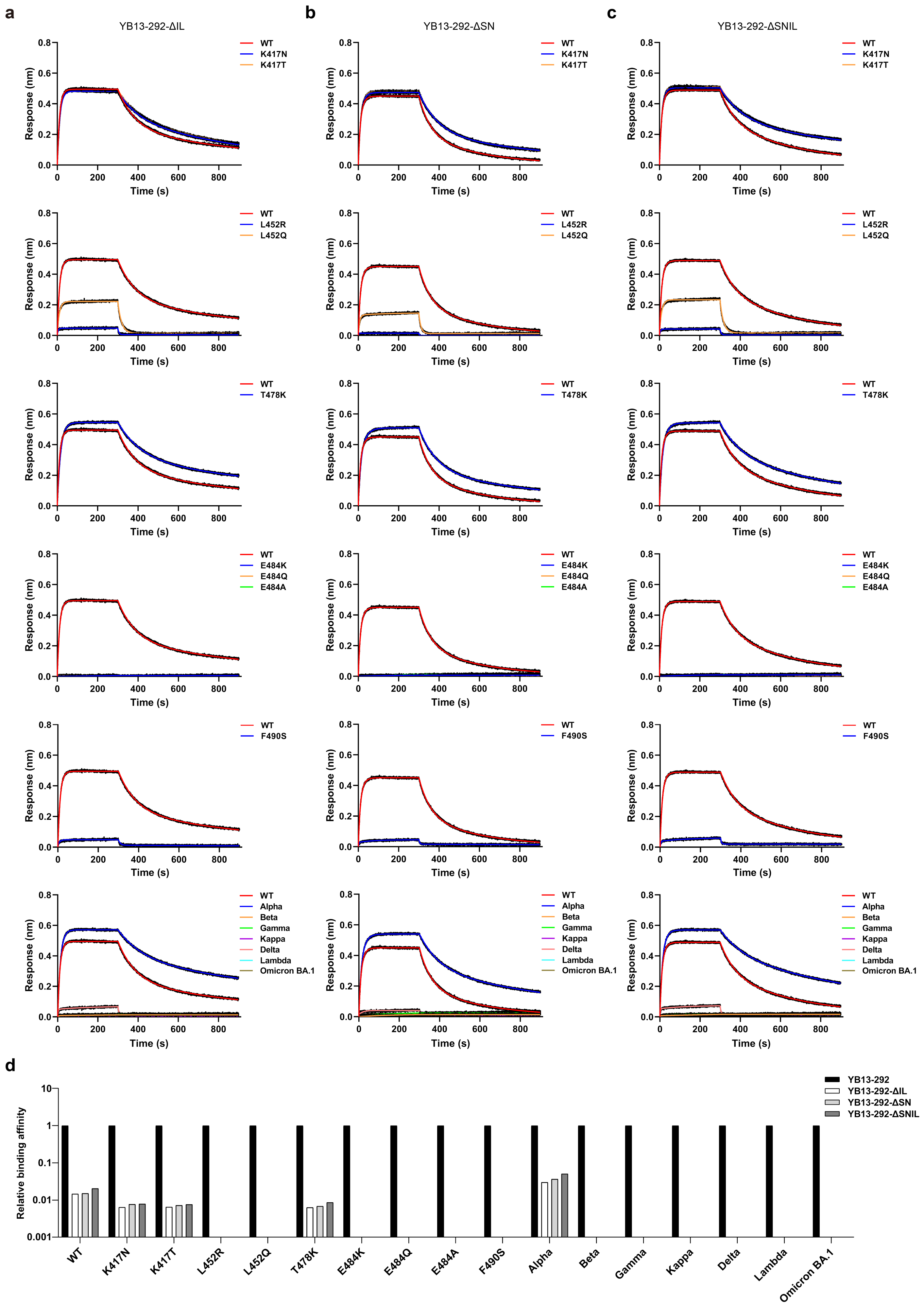

a
YB13-292-ΔIL
b
YB13-292-ΔSN
c
YB13-292-ΔSNIL
Response (nm)
Time (s)
WT
K417N
K417T
L452R
L452Q
T478K
E484K
E484Q
E484A
F490S
Alpha
Beta
Gamma
Kappa
Delta
Lambda
Omicron BA.1
d
Relative binding affinity
YB13-292
YB13-292-ΔIL
YB13-292-ΔSN
YB13-292-ΔSNIL

Supplementary Fig. 11 | Effect of the HCDR2 "SNIL" insertion on YB13-292 antigen binding. a-c, Different variants of YB13-292 antibody were generated, including variations of the "SNIL" insertion (YB13-292-ΔIL, YB13-292-ΔSN) or YB13-292 without the "SNIL" insertion (YB13-292-ΔSNIL). They were immobilized onto BLI Protein A biosensors and their binding to different RBDs (RBD point mutants or VOC RBDs at 200 nM) were assayed. Association and dissociation rates (k_{on}, k_{off}) were calculated by curve fitting, dissociation constants (K_D) were calculated as k_{off}/k_{on}, these kinetic parameters are present in **Supplementary Table 4**. **d**, Fold changes in dissociation constants (K_D) affected by different "SNIL" insertion variations for YB13-292 are shown. These are calculated by normalization to K_Ds calculated from binding curves of unmodified YB13-292 (K_D data used for comparisons are shown in **Supplementary Tables 3 and 4**).

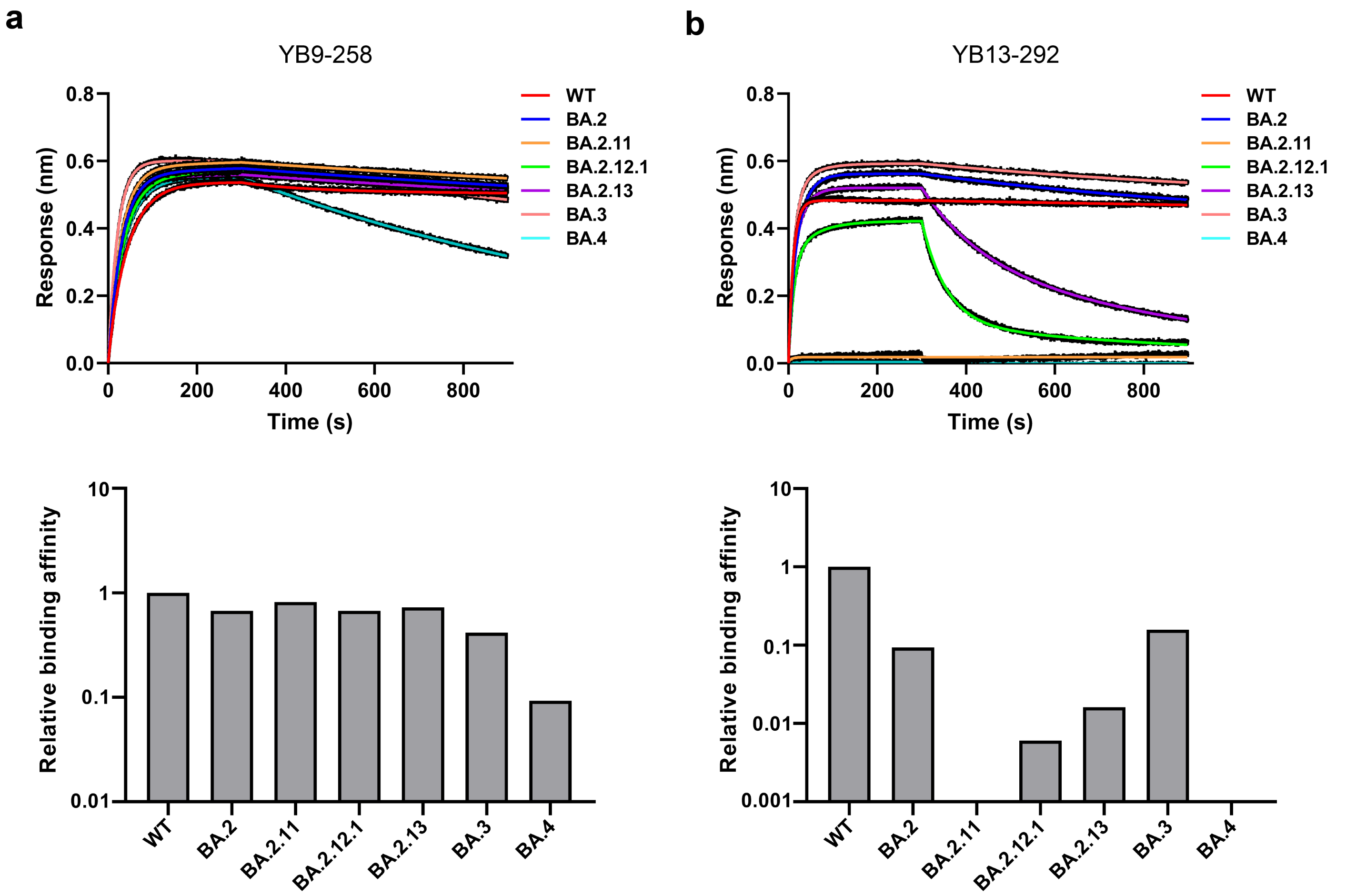

Supplementary Fig. 12 | Binding of YB9-258 and YB13-292 to Omicron subvariant RBDs. **a**, YB9-258, **b**, YB13-292 were immobilized onto Protein A biosensors. Wildtype RBD and RBDs of indicated Omicron subvariants were diluted to 200 nM. Sensors immobilized with IgGs were submerged into RBD solutions to record antigen association and dissociation. Association and dissociation rates (k_{on}, k_{off}) were calculated by curve fitting, dissociation constants (K_D) were calculated as k_{off}/k_{on}, these kinetic parameters are present in **Supplementary Table 5**. Fold changes in dissociation constants (K_D) affected by different Omicron variant RBDs for YB9-258 and YB13-292 are shown in lower panels. These are calculated by normalization to K_Ds calculated from binding reactions of YB9-258 and YB13-292 to wildtype RBD.

Supplementary Tables

Supplementary Table 1 | Patient sample information

Group	Sample pool	Patient (Pt)	Gender	Age	Infected virus strain	Disease severity	Symptom	Vaccine	Dose of vaccine	Duration of hospitalization (days)	Samples collection time (days after discharge)	IgG (WT)	Antigen of sorting	Re-covery cells
Non-vaccinated	W1#	Pt1	Male	30-40	WT	Mild	Fever, cough	NA	0	15	2	8	WT-RBD	5762
		Pt2	Male	30-40	WT	Mild	Asymptomatic	NA	0	14	13	8		
	W2#	Pt3	Male	50-60	WT	Mild	Headache, fever, cough, shortness of breath	NA	0	13	14	35		1221
	W4#	Pt5	Female	40-50	WT	Mild	Asymptomatic	NA	0	16	2	190		2577
	W5#	Pt6	Male	30-40	WT	Mild	Fever, cough, shortness of breath	NA	0	11	2	258		2215
	W6#	Pt7	Male	20-30	WT	Mild	Asymptomatic	NA	0	9	2	88		435
		Pt8	Male	30-40	WT	Mild	Fever, cough	NA	0	14	2	111		
	W7#	Pt9	Female	20-30	WT	Mild	Asymptomatic	NA	0	9	7	125		270
		Pt10	Male	40-50	WT	Mild	Asymptomatic	NA	0	11	2	87		
		Pt11	Male	50-60	WT	Mild	Cough	NA	0	15	2	91		
		Pt12	Female	40-50	WT	Mild	Fever, cough	NA	0	20	12	348		
Delta-infected	YB9#	Pt57	Male	50-60	Delta	Mild	Cough	Sinovac	1	14	14	86	Delta-RBD&S1	4293
		Pt58	Female	60-70	Delta	Mild	Headache, cough, shortness of breath, fever	NA	0	15	14	169		
		Pt59	Female	40-50	Delta	Mild	Fever, cough	Sinovac	1	17	14	172		
	YB12#	Pt77	Male	30-40	Delta	Moderate	Fever, cough	Sinopharm	2	15	5	421		1099
		Pt78	Female	20-30	Delta	Mild	Asymptomatic	NA	0	13	4	22		
		Pt79	Male	20-30	Delta	Mild	Asymptomatic	Sinopharm	2	14	4	109		
		Pt80	Male	20-30	Delta	Moderate	Fever, cough, shortness of breath	Sinopharm	2	11	4	463		
	YB13#	Pt81	Male	30-40	Delta	Moderate	Fever, cough, shortness of breath	Sinopharm	1	14	5	54		1086
		Pt82	Male	30-40	Delta	Mild	Asymptomatic	Sinopharm	2	13	4	86		
		Pt83	Male	40-50	Delta	Mild	Chest distress	Sinopharm	1	16	6	156		
		Pt84	Male	40-50	Delta	Mild	Asymptomatic	Sinopharm	2	17	4	268		
	YB14#	Pt85	Male	50-60	Delta	Mild	Fever, cough, chest distress	Sinopharm	2	11	5	501		404
		Pt86	Male	50-60	Delta	Mild	Asymptomatic	Sinopharm	2	11	7	73		
		Pt87	Male	50-60	Delta	Mild	Asymptomatic	Sinovac	1	11	4	240		
		Pt88	Male	40-50	Delta	Mild	Cough	Sinopharm	2	13	12	2		

NA: no vaccine

Supplementary Table 2 | Summary of rate constants (k_{on}, k_{off}) and dissociation constants (K_D) for the antibody interactions in Fig. 2b

	YB9-120			YB12-197			YB13-208			YB9-258			YB13-281			YB13-292		
	k_{on} (M^{-1} s^{-1})	k_{off} (s^{-1})	K_D (nM)	k_{on} (M^{-1} s^{-1})	k_{off} (s^{-1})	K_D (nM)	k_{on} (M^{-1} s^{-1})	k_{off} (s^{-1})	K_D (nM)	k_{on} (M^{-1} s^{-1})	k_{off} (s^{-1})	K_D (nM)	k_{on} (M^{-1} s^{-1})	k_{off} (s^{-1})	K_D (nM)	k_{on} (M^{-1} s^{-1})	k_{off} (s^{-1})	K_D (nM)
**S-R/Wildtype**	3.71×10^{4} (k_{on1})	4.31×10^{-5} (k_{off1})	1.16 (k_{off1}/k_{on1})	1.97×10^{4} (k_{on1})	1.60×10^{-4} (k_{off1})	8.1 (k_{off1}/k_{on1})	3.79×10^{4} (k_{on1})	3.16×10^{-5} (k_{off1})	0.83 (k_{off1}/k_{on1})	8.93×10^{3} (k_{on1})	3.42×10^{-6} (k_{off1})	0.38 (k_{off1}/k_{on1})	2.15×10^{4} (k_{on1})	3.01×10^{-4} (k_{off1})	14.01 (k_{off1}/k_{on1})	3.52×10^{4} (k_{on1})	1.18×10^{-4} (k_{off1})	3.34 (k_{off1}/k_{on1})
	2.02×10^{5} (k_{on2})	$< 1.00\times10^{-7}$ (k_{off2})	0.21 (k_{off1}/k_{on2})	4.07×10^{4} (k_{on2})	$< 1.00\times10^{-7}$ (k_{off2})	3.92 (k_{off1}/k_{on2})	2.55×10^{5} (k_{on2})	1.86×10^{-5} (k_{off2})	0.12 (k_{off1}/k_{on2})	9.12×10^{3} (k_{on2})	$< 1.00\times10^{-7}$ (k_{off2})	0.38 (k_{off1}/k_{on2})	2.61×10^{4} (k_{on2})	$< 1.00\times10^{-7}$ (k_{off2})	11.54 (k_{off1}/k_{on2})	2.40×10^{5} (k_{on2})	5.23×10^{-5} (k_{off2})	0.49 (k_{off1}/k_{on2})
			< 0.0027 (k_{off2}/k_{on1})			< 0.0051 (k_{off2}/k_{on1})			0.49 (k_{off2}/k_{on1})			< 0.011 (k_{off2}/k_{on1})			< 0.0046 (k_{off2}/k_{on1})			1.49 (k_{off2}/k_{on1})
									0.073 (k_{off2}/k_{on2})									0.22 (k_{off2}/k_{on2})
**S-R/Delta**	2.96×10^{4} (k_{on1})	3.30×10^{-5} (k_{off1})	1.12 (k_{off1}/k_{on1})	1.71×10^{4} (k_{on1})	2.68×10^{-7} (k_{off1})	$\leq$0.016 (k_{off1}/k_{on1})	4.19×10^{4} (k_{on1})	7.30×10^{-5} (k_{off1})	1.74 (k_{off1}/k_{on1})	4.76×10^{3} (k_{on1})	3.48×10^{-7} (k_{off1})	$\leq$0.073 (k_{off1}/k_{on1})	2.49×10^{4} (k_{on1})	1.28×10^{-4} (k_{off1})	5.12 (k_{off1}/k_{on1})	2.75×10^{4} (k_{on1})	8.62×10^{-5} (k_{off1})	3.14 (k_{off1}/k_{on1})
	1.66×10^{5} (k_{on2})	$< 1.00\times10^{-7}$ (k_{off2})	0.2 (k_{off1}/k_{on2})	2.73×10^{5} (k_{on2})	$< 1.00\times10^{-7}$ (k_{off2})		3.41×10^{5} (k_{on2})	1.04×10^{-5} (k_{off2})	0.21 (k_{off1}/k_{on2})	2.04×10^{4} (k_{on2})	$< 1.00\times10^{-7}$ (k_{off2})		2.89×10^{4} (k_{on2})	$< 1.00\times10^{-7}$ (k_{off2})	4.42 (k_{off1}/k_{on2})	2.32×10^{5} (k_{on2})	7.13×10^{-5} (k_{off2})	0.37 (k_{off1}/k_{on2})
			< 0.0034 (k_{off2}/k_{on1})						0.25 (k_{off2}/k_{on1})						< 0.004 (k_{off2}/k_{on1})			2.6 (k_{off2}/k_{on1})
									0.03 (k_{off2}/k_{on2})									0.31 (k_{off2}/k_{on2})
**S-R/Omicron BA.1**	1.11×10^{4} (k_{on1})	5.98×10^{-6} (k_{off1})	0.54 (k_{off1}/k_{on1})	1.23×10^{4} (k_{on1})	1.04×10^{-4} (k_{off1})	8.45 (k_{off1}/k_{on1})	2.57×10^{4} (k_{on1})	4.32×10^{-3} (k_{off1})	168 (k_{off1}/k_{on1})	7.28×10^{3} (k_{on1})	3.72×10^{-7} (k_{off1})	$\leq$0.051 (k_{off1}/k_{on1})	1.01×10^{4} (k_{on1})	$< 1.00\times10^{-7}$ (k_{off1})	< 0.0099 (k_{off1}/k_{on1})	1.65×10^{4} (k_{on1})	5.50×10^{-5} (k_{off1})	3.33 (k_{off1}/k_{on1})
	5.51×10^{4} (k_{on2})	$< 1.00\times10^{-7}$ (k_{off2})	0.11 (k_{off1}/k_{on2})	1.25×10^{4} (k_{on2})	$< 1.00\times10^{-7}$ (k_{off2})	8.34 (k_{off1}/k_{on2})	8.71×10^{4} (k_{on2})	1.94×10^{-5} (k_{off2})	49.55 (k_{off1}/k_{on2})	1.15×10^{4} (k_{on2})	$< 1.00\times10^{-7}$ (k_{off2})		1.02×10^{4} (k_{on2})	$< 1.00\times10^{-7}$ (k_{off2})		1.29×10^{5} (k_{on2})	3.55×10^{-5} (k_{off2})	0.43 (k_{off1}/k_{on2})
			< 0.009 (k_{off2}/k_{on1})			< 0.0081 (k_{off2}/k_{on1})			0.75 (k_{off2}/k_{on1})									2.15 (k_{off2}/k_{on1})
									0.22 (k_{off2}/k_{on2})									0.28 (k_{off2}/k_{on2})
**S-R/Omicron BA.2**	1.75×10^{4} (k_{on1})	7.63×10^{-7} (k_{off1})	$\leq$0.044 (k_{off1}/k_{on1})	7.52×10^{3} (k_{on1})	6.64×10^{-5} (k_{off1})	8.83 (k_{off1}/k_{on1})	6.90×10^{3} (k_{on1})	2.08×10^{-5} (k_{off1})	3.02 (k_{off1}/k_{on1})	2.02×10^{4} (k_{on1})	5.52×10^{-6} (k_{off1})	0.27 (k_{off1}/k_{on1})	1.03×10^{4} (k_{on1})	4.70×10^{-6} (k_{off1})	0.46 (k_{off1}/k_{on1})	5.34×10^{4} (k_{on1})	4.68×10^{-6} (k_{off1})	$\leq$0.088 (k_{off1}/k_{on1})
	4.93×10^{4} (k_{on2})	$< 1.00\times10^{-7}$ (k_{off2})		8.32×10^{3} (k_{on2})	$< 1.00\times10^{-7}$ (k_{off2})	7.98 (k_{off1}/k_{on2})	8.34×10^{4} (k_{on2})	1.02×10^{-7} (k_{off2})	0.25 (k_{off1}/k_{on2})	2.19×10^{4} (k_{on2})	$< 1.00\times10^{-7}$ (k_{off2})	0.25 (k_{off1}/k_{on2})	1.07×10^{4} (k_{on2})	$< 1.00\times10^{-7}$ (k_{off2})	0.44 (k_{off1}/k_{on2})	5.71×10^{4} (k_{on2})	$< 1.00\times10^{-7}$ (k_{off2})	
						< 0.013 (k_{off2}/k_{on1})			$\leq$0.015 (k_{off2}/k_{on1})			< 0.005 (k_{off2}/k_{on1})			< 0.0097 (k_{off2}/k_{on1})			
**S-R/Omicron BA.3**	1.71×10^{4} (k_{on1})	1.69×10^{-6} (k_{off1})	$\leq$0.099 (k_{off1}/k_{on1})	1.99×10^{4} (k_{on1})	2.11×10^{-4} (k_{off1})	10.61 (k_{off1}/k_{on1})	1.90×10^{4} (k_{on1})	5.25×10^{-4} (k_{off1})	27.64 (k_{off1}/k_{on1})	1.59×10^{4} (k_{on1})	1.78×10^{-6} (k_{off1})	0.11 (k_{off1}/k_{on1})	1.80×10^{4} (k_{on1})	1.71×10^{-4} (k_{off1})	9.48 (k_{off1}/k_{on1})	2.76×10^{4} (k_{on1})	4.60×10^{-7} (k_{off1})	$\leq$0.017 (k_{off1}/k_{on1})
	2.44×10^{4} (k_{on2})	$< 1.00\times10^{-7}$ (k_{off2})		3.50×10^{4} (k_{on2})	$< 1.00\times10^{-7}$ (k_{off2})	6.04 (k_{off1}/k_{on2})	2.65×10^{4} (k_{on2})	$< 1.00\times10^{-7}$ (k_{off2})	19.81 (k_{off1}/k_{on2})	2.87×10^{4} (k_{on2})	$< 1.00\times10^{-7}$ (k_{off2})	$\leq$0.062 (k_{off1}/k_{on2})	2.08×10^{4} (k_{on2})	$< 1.00\times10^{-7}$ (k_{off2})	8.24 (k_{off1}/k_{on2})	1.55×10^{5} (k_{on2})	$< 1.00\times10^{-7}$ (k_{off2})	
						< 0.005 (k_{off2}/k_{on1})			< 0.0053 (k_{off2}/k_{on1})						< 0.0055 (k_{off2}/k_{on1})			
**S-R/Omicron BA.4**	3.16×10^{4} (k_{on1})	1.41×10^{-6} (k_{off1})	$\leq$0.045 (k_{off1}/k_{on1})	8.40×10^{3} (k_{on1})	7.98×10^{-6} (k_{off1})	0.95 (k_{off1}/k_{on1})	2.99×10^{4} (k_{on1})	6.22×10^{-5} (k_{off1})	2.08 (k_{off1}/k_{on1})	1.17×10^{4} (k_{on1})	2.48×10^{-7} (k_{off1})	$\leq$0.021 (k_{off1}/k_{on1})	1.48×10^{4} (k_{on1})	4.11×10^{-5} (k_{off1})	2.78 (k_{off1}/k_{on1})	2.15×10^{4} (k_{on1})	3.29×10^{-4} (k_{off1})	15.3 (k_{off1}/k_{on1})
	3.29×10^{4} (k_{on2})	$< 1.00\times10^{-7}$ (k_{off2})		1.76×10^{4} (k_{on2})	$< 1.00\times10^{-7}$ (k_{off2})	0.45 (k_{off1}/k_{on2})	1.62×10^{5} (k_{on2})	$< 1.00\times10^{-7}$ (k_{off2})	0.39 (k_{off1}/k_{on2})	2.21×10^{4} (k_{on2})	$< 1.00\times10^{-7}$ (k_{off2})		1.90×10^{4} (k_{on2})	$< 1.00\times10^{-7}$ (k_{off2})	2.16 (k_{off1}/k_{on2})	3.01×10^{4} (k_{on2})	$< 1.00\times10^{-7}$ (k_{off2})	10.91 (k_{off1}/k_{on2})
						< 0.012 (k_{off2}/k_{on1})			< 0.0033 (k_{off2}/k_{on1})						< 0.0068 (k_{off2}/k_{on1})			< 0.0047 (k_{off2}/k_{on1})

Supplementary Table 3 | Kinetic parameters of YB9-258, YB13-292 and 47D1 binding to RBD mutants in Supplementary Fig. 3

	YB9-258			YB13-292			47D1		
	k_{on} (M^{-1} s^{-1})	k_{off} (s^{-1})	K_D (nM)	k_{on} (M^{-1} s^{-1})	k_{off} (s^{-1})	K_D (nM)	k_{on} (M^{-1} s^{-1})	k_{off} (s^{-1})	K_D (nM)
WT	1.21×10^{5}	7.70×10^{-5}	0.63	5.00×10^{5}	1.97×10^{-4}	0.39	9.98×10^{5}	4.10×10^{-3}	4.11
K417N	7.58×10^{4}	2.18×10^{-4}	2.88	5.05×10^{5}	9.84×10^{-5}	0.19	5.88×10^{5}	2.06×10^{-3}	3.5
K417T	7.85×10^{4}	7.30×10^{-5}	0.93	4.93×10^{5}	9.69×10^{-5}	0.2			
L452R	1.25×10^{5}	7.81×10^{-5}	0.63	2.70×10^{5}	2.36×10^{-3}	8.73	8.92×10^{5}	6.00×10^{-2}	67.29
L452Q	9.75×10^{4}	6.94×10^{-5}	0.71	3.33×10^{5}	8.43×10^{-5}	0.25			
T478K	1.01×10^{5}	4.20×10^{-5}	0.42	3.34×10^{5}	7.03×10^{-5}	0.21	6.23×10^{5}	2.76×10^{-3}	4.43
E484K	1.41×10^{5}	7.21×10^{-5}	0.51	1.18×10^{5}	5.16×10^{-3}	43.71	-	-	no binding
E484Q	1.34×10^{5}	8.17×10^{-5}	0.61	3.92×10^{5}	7.36×10^{-4}	1.88	5.70×10^{5}	7.99×10^{-2}	140
E484A	1.37×10^{5}	7.47×10^{-5}	0.54	2.35×10^{5}	2.28×10^{-3}	9.73			
F490S	1.03×10^{5}	7.68×10^{-5}	0.74	1.75×10^{5}	2.86×10^{-3}	16.38	-	-	no binding
Alpha	1.28×10^{5}	4.75×10^{-5}	0.37	4.07×10^{5}	3.05×10^{-4}	0.75	2.15×10^{6}	5.47×10^{-3}	2.55
Beta	1.15×10^{5}	2.60×10^{-4}	2.26	3.19×10^{5}	3.25×10^{-3}	10.21	-	-	no binding
Gamma	1.16×10^{5}	7.10×10^{-5}	0.61	2.72×10^{5}	2.43×10^{-3}	8.97			
Kappa	1.19×10^{5}	4.73×10^{-5}	0.4	-	-	no binding	-	-	no binding
Delta	1.20×10^{5}	5.82×10^{-5}	0.49	4.09×10^{5}	1.12×10^{-3}	2.74	6.61×10^{5}	4.04×10^{-2}	61.1
Lambda	1.11×10^{5}	1.16×10^{-4}	1.04	2.19×10^{5}	1.08×10^{-1}	494			
Omicron BA.1	4.73×10^{4}	3.32×10^{-4}	7.02	1.64×10^{5}	2.81×10^{-3}	17.13			

Supplementary Table 4 | Summary of rate constants (*k_{on}*, *k_{off}*) and dissociation constants (*K_D*) for the bindings between YB13-292 variants and RBD mutants in Supplementary Fig. 11

	YB13-292-ΔIL			YB13-292-ΔSN			YB13-292-ΔSNIL		
	k_{on} (M^{-1} s^{-1})	k_{off} (s^{-1})	K_D (nM)	k_{on} (M^{-1} s^{-1})	k_{off} (s^{-1})	K_D (nM)	k_{on} (M^{-1} s^{-1})	k_{off} (s^{-1})	K_D (nM)
WT	3.89×10^{5}	1.19×10^{-2}	30.66	5.31×10^{5}	1.58×10^{-2}	29.7	5.34×10^{5}	1.16×10^{-2}	21.8
K417N	3.53×10^{5}	1.05×10^{-2}	29.76	4.21×10^{5}	1.04×10^{-2}	24.64	3.78×10^{5}	9.06×10^{-3}	23.95
K417T	3.46×10^{6}	1.07×10^{-2}	30.84	4.19×10^{5}	1.16×10^{-2}	27.65	3.75×10^{5}	9.75×10^{-3}	26.03
L452R	-	-	no binding	-	-	no binding	-	-	no binding
L452Q	1.83×10^{5}	5.54×10^{-2}	303	2.67×10^{5}	9.84×10^{-2}	368	1.58×10^{5}	6.03×10^{-2}	383
T478K	2.59×10^{5}	8.66×10^{-3}	33.39	3.79×10^{5}	1.16×10^{-2}	30.51	4.11×10^{5}	9.92×10^{-3}	24.14
E484K	-	-	no binding	-	-	no binding	-	-	no binding
E484Q	-	-	no binding	-	-	no binding	-	-	no binding
E484A	-	-	no binding	-	-	no binding	-	-	no binding
F490S	-	-	no binding	-	-	no binding	-	-	no binding
Alpha	3.22×10^{5}	8.05×10^{-3}	25.03	4.99×10^{5}	1.02×10^{-2}	20.43	5.60×10^{5}	8.28×10^{-3}	14.78
Beta	-	-	no binding	-	-	no binding	-	-	no binding
Gamma	-	-	no binding	-	-	no binding	-	-	no binding
Kappa	-	-	no binding	-	-	no binding	-	-	no binding
Delta	-	-	no binding	-	-	no binding	-	-	no binding
Lambda	-	-	no binding	-	-	no binding	-	-	no binding
Omicron BA.1	-	-	no binding	-	-	no binding	-	-	no binding

Supplementary Table 5 | Kinetic parameters of YB9-258 and YB13-292 binding to Omicron subvariant RBDs in Supplementary Fig. 12

	YB9-258			YB13-292		
	k_{on} (M^{-1} s^{-1})	k_{off} (s^{-1})	K_D (nM)	k_{on} (M^{-1} s^{-1})	k_{off} (s^{-1})	K_D (nM)
WT	1.21×10^5	7.70×10^{-5}	0.63	5.00×10^5	1.97×10^{-4}	0.39
BA.2	1.56×10^5	1.47×10^{-4}	0.94	2.62×10^5	1.09×10^{-3}	4.17
BA.2.11	1.67×10^5	1.29×10^{-4}	0.77	-	-	no binding
BA.2.12.1	1.36×10^5	1.29×10^{-4}	0.94	2.76×10^5	1.78×10^{-2}	64.5
BA.2.13	1.67×10^5	1.45×10^{-4}	0.87	3.13×10^5	7.57×10^{-3}	24.17
BA.3	2.40×10^5	3.66×10^{-4}	1.52	4.24×10^5	1.05×10^{-3}	2.47
BA.4	1.35×10^5	9.16×10^{-4}	6.81	-	-	no binding

Supplementary Table 6 | Cryo-EM data collection, refinement and validation statistics

	Omicron BA.1 S-6P:YB9-258 Fab/1 RBD "up"<br>(EMD-34649, PDB 8HC2)	Omicron BA.1 S-6P:YB9-258 Fab/2 RBD "up"<br>(EMD-34650, PDB 8HC3)	Wildtype S-6P:YB9-258 Fab:R1-32 Fab/3 RBD "up"<br>(EMD-34651, PDB 8HC4)	Wildtype S1:YB9-258 Fab:R1-32 Fab<br>(EMD-34652, PDB 8HC5)	Omicron BA.1 S-6P:YB9-258 Fab/Fab focused<br>(EMD-34653, PDB 8HC6)
**Data collection and processing**					
Magnification	45000	45000	165000	165000	45000
Voltage (kV)	200	200	300	300	200
Electron exposure ($e^{-}/Å^2$)	60	60	50	50	60
Defocus range (μm)	0.8-2.5	0.8-2.5	0.6-2.0	0.6-2.0	0.8-2.5
Pixel size (Å)	0.88	0.88	0.732	0.732	0.88
Movies (no.)	11470	11470	12840	12840	11470
Initial particle images (no.)	1543443	1543443	1873738	1873738	1543443
Symmetry imposed	*C1*	*C1*	*C1*	*C1*	*C1*
Final particle images (no.)	70195	287435	95333	202540	183769
Map resolution (Å)	6.21	4.31	3.54	3.43	4.69
FSC threshold	0.143	0.143	0.143	0.143	0.143
Map resolution range (Å)	4.8-17.8	3.9-14.4	3.2-17.8	3.3-11.0	4.4-12.3
**Refinement**					
Initial model used	PDB 7T9K	PDB 7T9K	PDB 7A98	PDB 7A98	PDB 7T9K
Model resolution (Å)	7.36	4.55	8.02	3.89	5.51
FSC threshold	0.5	0.5	0.5	0.5	0.5
Map sharpening *B* factor ($Å^2$)	-165	-143	-74	-82	-130
Model composition					
Non-hydrogen atoms	28278	31497	45096	11400	7377
Protein residues	3525	3954	5805	1493	933
Ligands	54	54	54	1	7
B factors ($Å^2$)					
Protein	372.19	222.45	21.16	26.32	222.53
Ligand	325.64	183.87	19.01	6.41	200.06
R.m.s. deviations					
Bond lengths (Å)	0.002	0.002	0.002	0.002	0.002
Bond angles (°)	0.534	0.559	0.497	0.525	0.545
**Validation**					
MolProbity score	1.47	1.53	1.55	1.56	1.57
Clashscore	5.15	5.53	5.29	5.34	4.81
Poor rotamers (%)	0.06	0.00	0.04	0.00	0.12
Ramachandran plot					
Favored (%)	96.77	96.53	96.04	96.01	95.40
Allowed (%)	3.23	3.47	3.96	3.99	4.60
Disallowed (%)	0.00	0.00	0.00	0.00	0.00

	Omicron BA.1 S-6P:YB9-258 Fab/RBD-dimer focused (EMD-34654, PDB 8HC7)	Omicron BA.1 S-6P:YB13-292 Fab/0 RBD "up" (EMD-34656, PDB 8HC9)	Omicron BA.1 S-6P:YB13-292 Fab/1 RBD "up" (EMD-34657, PDB 8HCA)	Omicron BA.1 S-6P:YB13-292 Fab/2 RBD "up" (EMD-34658, PDB 8HCB)	Omicron BA.1 S-6P:YB13-292 Fab/Fab focused (EMD-34655, PDB 8HC8)
**Data collection and processing**					
Magnification	45000	45000	45000	45000	45000
Voltage (kV)	200	200	200	200	200
Electron exposure ($e^{-}/Å^2$)	60	60	60	60	60
Defocus range (μm)	0.8-2.5	0.8-2.5	0.8-2.5	0.8-2.5	0.8-2.5
Pixel size (Å)	0.88	0.88	0.88	0.88	0.88
Movies (no.)	11470	4485	4485	4485	4485
Initial particle images (no.)	1543443	875728	875728	875728	875728
Symmetry imposed	*C1*	*C1*	*C1*	*C1*	*C1*
Final particle images (no.)	287435	36241	103226	166815	210698
Map resolution (Å)	4.54	6.03	4.35	4.18	3.95
FSC threshold	0.143	0.143	0.143	0.143	0.143
Map resolution range (Å)	4.3-12.2	4.4-19.4	3.9-17.5	3.7-13.9	3.8-10.0
**Refinement**					
Initial model used	PDB 7T9K	PDB 7T9K	PDB 7T9K	PDB 7T9K	PDB 7T9K
Model resolution (Å)	4.84	7.39	4.55	4.30	4.13
FSC threshold	0.5	0.5	0.5	0.5	0.5
Map sharpening *B* factor ($Å^2$)	-164	-151	-103	-97	-115
Model composition					
Non-hydrogen atoms	7373	35187	35187	35187	4839
Protein residues	919	4449	4449	4449	628
Ligands	7	54	54	54	1
B factors ($Å^2$)					
Protein	167.27	369.35	187.73	151.19	31.37
Ligand	133.65	256.26	133.21	128.04	88.27
R.m.s. deviations					
Bond lengths (Å)	0.002	0.002	0.002	0.002	0.003
Bond angles (°)	0.588	0.566	0.564	0.573	0.706
**Validation**					
MolProbity score	1.64	1.61	1.64	1.62	1.60
Clashscore	5.72	6.05	5.79	5.69	5.70
Poor rotamers (%)	0.00	0.03	0.44	0.39	0.56
Ramachandran plot					
Favored (%)	95.33	95.96	95.41	95.48	95.81
Allowed (%)	4.67	4.04	4.59	4.49	4.19
Disallowed (%)	0.00	0.00	0.00	0.00	0.00

Supplementary References

1 Yin, W. *et al.* Structures of the Omicron spike trimer with ACE2 and an anti-Omicron antibody. *Science* **375**, 1048-1053, doi:10.1126/science.abn8863 (2022).

2 He, P. *et al.* SARS-CoV-2 Delta and Omicron variants evade population antibody response by mutations in a single spike epitope. *Nat Microbiol* **7**, 1635-1649, doi:10.1038/s41564-022-01235-4 (2022).

3 Krissinel, E. & Henrick, K. Inference of macromolecular assemblies from crystalline state. *J Mol Biol* **372**, 774-797, doi:10.1016/j.jmb.2007.05.022 (2007).
