# Supplementary material for: Somatically hypermutated antibodies isolated from SARS-CoV-2 Delta infected patients cross-neutralize heterologous variants
Source: Nat Commun. 2023 Feb 24;14:1058. doi: 10.1038/s41467-023-36761-0 (PMC9951844; doi:10.1038/s41467-023-36761-0)
Supplement: Supplementary file 3 — Description of Additional Supplementary Files [file 41467_2023_36761_MOESM3_ESM.pdf]

Description of Additional Supplementary Files

Supplementary Data 1: Characteristics of the 117 selected antibodies.

Supplementary Data 2: Binding affinity, neutralization and sequence features of the 22 selected antibodies.
