# Supplementary material for: Somatically hypermutated antibodies isolated from SARS-CoV-2 Delta infected patients cross-neutralize heterologous variants
Source: Nat Commun. 2023 Feb 24;14:1058. doi: 10.1038/s41467-023-36761-0 (PMC9951844; doi:10.1038/s41467-023-36761-0)
Supplement: Supplementary file 6 — Reporting Summary [file 41467_2023_36761_MOESM6_ESM.pdf]

nature portfolio

Corresponding author(s): Xiaoli Xiong
Haisheng Yu

Last updated by author(s): Jan 12, 2023

Reporting Summary

Nature Portfolio wishes to improve the reproducibility of the work that we publish. This form provides structure for consistency and transparency in reporting. For further information on Nature Portfolio policies, see our Editorial Policies and the Editorial Policy Checklist.

Statistics

For all statistical analyses, confirm that the following items are present in the figure legend, table legend, main text, or Methods section.

n/a	Confirmed	
☐	☒	The exact sample size (*n*) for each experimental group/condition, given as a discrete number and unit of measurement
☐	☒	A statement on whether measurements were taken from distinct samples or whether the same sample was measured repeatedly
☐	☒	The statistical test(s) used AND whether they are one- or two-sided<br>*Only common tests should be described solely by name; describe more complex techniques in the Methods section.*
☒	☐	A description of all covariates tested
☒	☐	A description of any assumptions or corrections, such as tests of normality and adjustment for multiple comparisons
☐	☒	A full description of the statistical parameters including central tendency (e.g. means) or other basic estimates (e.g. regression coefficient) AND variation (e.g. standard deviation) or associated estimates of uncertainty (e.g. confidence intervals)
☐	☒	For null hypothesis testing, the test statistic (e.g. *F*, *t*, *r*) with confidence intervals, effect sizes, degrees of freedom and *P* value noted<br>*Give P values as exact values whenever suitable.*
☒	☐	For Bayesian analysis, information on the choice of priors and Markov chain Monte Carlo settings
☒	☐	For hierarchical and complex designs, identification of the appropriate level for tests and full reporting of outcomes
☒	☐	Estimates of effect sizes (e.g. Cohen's *d*, Pearson's *r*), indicating how they were calculated

Our web collection on statistics for biologists contains articles on many of the points above.

Software and code

Policy information about availability of computer code

Data collection	SPR binding assays collected and analyzed using Biacore Evaluation and Biacore Insight software;<br>SerialEM version 3.8.7 and EPU version 2.14.0 automated image acquisition software;<br>Fortebio Octet RED96 instrument software (available and referenced in the Methods section).
Data analysis	Flow cytometry data were analyzed using FlowJo v10.<br>ELISA, Binding and Neutralization data were analyzed using Graphpad Prism 8.0 as described in Methods.<br>CryoEM data processing (all available and referenced in methods): RELION v4.0; MotionCor2 algorithm; Warp 1.0.7.<br>Modelling/structure refinement/visualization (all available and referenced in methods): Coot v0.9.6; PHENIX 1.20.1; UCSF Chimera 1.14; PDBe PISA v1.52.<br>Single cell sequencing data analysis: Cell Ranger v6.1.2; R statistical software v4.1.2; Seurat v4.0.5; SingleR vl.8.0; IgBlast vl.18.0; Biostrings V2.60.2.<br>Kinetic constants calculating: Fortebio Octet Data Analysis Software HT v12.0.2.59.<br>Polyreactivity data analysis: Graphpad Prism 8.0.<br>Sequence alignment: IMGT/V-QUEST (http://imgt.org).

For manuscripts utilizing custom algorithms or software that are central to the research but not yet described in published literature, software must be made available to editors and reviewers. We strongly encourage code deposition in a community repository (e.g. GitHub). See the Nature Portfolio guidelines for submitting code & software for further information.

Data

Policy information about availability of data

All manuscripts must include a data availability statement. This statement should provide the following information, where applicable:

- Accession codes, unique identifiers, or web links for publicly available datasets
- A description of any restrictions on data availability
- For clinical datasets or third party data, please ensure that the statement adheres to our policy

The raw sequencing data has been deposited in the Genome Sequence Archive of the BIG Data Center, Beijing Institute of Genomics (BIG), Chinese Academy of Science, under accession number PRJCA012020.
Databases used in this study include COV-AbDab database: http://opig.stats.ox.ac.uk/webapps/covabdab/.
Cryo-EM density maps for the SARS-CoV-2 Wildtype and Omicron BA.1 spikes in complex with YB9-258 Fab have been deposited in the Electron Microscopy Data Bank (EMDB, https://www.ebi.ac.uk/emdb/) with accession codes EMD-34649, EMD-34650, EMD-34651, EMD-34652, EMD-34653 and EMD-34654. The related atomic models have been deposited in Protein Data Bank (PDB, https://www.rcsb.org/) under accession codes 8HC2, 8HC3, 8HC4, 8HC5, 8HC6 and 8HC7, respectively. Cryo-EM density maps for the SARS-CoV-2 Omicron BA.1 spike in complex with YB13-292 Fab have been deposited in the Electron Microscopy Data Bank (EMDB) with accession codes EMD-34655, EMD-34656, EMD-34657 and EMD-34658. The related atomic models have been deposited in Protein Data Bank (PDB) under accession codes 8HC8, 8HC9, 8HCA and 8HCB.

Human research participants

Policy information about studies involving human research participants and Sex and Gender in Research.

Reporting on sex and gender	Donors are 8 males and 3 females with an average age of 39 in non-vaccinated group, 12 males and 3 females with an average age of 42 in Delta-infected group. We did not perform sex- and gender-based analyses, antibody response should not be affected by sex.
Population characteristics	Convalescent COVID-19 patients were selected randomly who were cared in Guangzhou Eighth People's Hospital, China. Donors are 8 males and 3 females with an average age of 39 in non-vaccinated group, 12 males and 3 females with an average age of 42 in Delta-infected group. Eligible participants were adults aged 24-61 years who were diagnosed with SARS-CoV-2 infection by RT-PCR.
Recruitment	The patients agreed to provide the specimen for detection, further diagnostic and scientific research when hospitalization. The requirement for participants to be free of symptoms for at least 14 days might have favored enrollment of participants that developed mild COVID-19 courses of infection during the first weeks of recruitment.
Ethics oversight	Ethics Committee of Guangzhou Eighth People's Hospital (No. 202001134 and 202115202).

Note that full information on the approval of the study protocol must also be provided in the manuscript.

Field-specific reporting

Please select the one below that is the best fit for your research. If you are not sure, read the appropriate sections before making your selection.

☒ Life sciences ☐ Behavioural & social sciences ☐ Ecological, evolutionary & environmental sciences

For a reference copy of the document with all sections, see nature.com/documents/nr-reporting-summary-flat.pdf

Life sciences study design

All studies must disclose on these points even when the disclosure is negative.

Sample size	For patient study, sample size is not applicable, because no sample size calculation was performed during the design of the study. Other assays were performed for multiple replicates, which meets the requirement for statistical analysis and is sufficient for a good technical reproducibility.
Data exclusions	No data were excluded.
Replication	Experimental assays were performed in biological duplicate or triplicate (or more) according to or exceeding standards in the field. We conducted all neutralization and antibody functional assays in biological duplicate, triplicate, or more, as indicated in relevant figure legends. In all cases, representative figure displays were appropriately replicated.
Randomization	Not applicable to this study, as we do not report experiments that use randomized data.
Blinding	The investigators were not blinded to allocation during experiments and outcome assessment. Data collection and analysis were performed by different team, the sample classification were replaced by marks during data analysis.

Reporting for specific materials, systems and methods

We require information from authors about some types of materials, experimental systems and methods used in many studies. Here, indicate whether each material, system or method listed is relevant to your study. If you are not sure if a list item applies to your research, read the appropriate section before selecting a response.

Materials & experimental systems

n/a	Involved in the study
☐	☒ Antibodies
☐	☒ Eukaryotic cell lines
☒	☐ Palaeontology and archaeology
☒	☐ Animals and other organisms
☒	☐ Clinical data
☒	☐ Dual use research of concern

Methods

n/a	Involved in the study
☒	☐ ChIP-seq
☐	☒ Flow cytometry
☒	☐ MRI-based neuroimaging

Antibodies

Antibodies used	Flow cytometry:<br>• PE anti-human CD27 antibody (BD Biosciences, Cat# 566944, Clone name: O323, 1:50 dilution);<br>• Streptavidin-Allophycocyanin (Biolegend, Cat# 405243, 1:20 dilution);<br>• Streptavidin-Fluorescein Isothiocyanate (Biolegend, Cat# 405202, 1:20 dilution).
Validation	The antibodies in our study are all commercial available and their specificity are well characterized by the manufactors and other users. All antibodies were validated and dilution optimized using positive cells (PBMC) before performance of the study. The information are included in the Methods section.

Eukaryotic cell lines

Policy information about cell lines and Sex and Gender in Research

Cell line source(s)	293T cells: ATCC, CRL-3216;<br>293T-ACE2 cells have been described by previously study (Feng et al., Nature Communications. 2020) and were provided by Dr. Ling Chen from Guangzhou Institutes of Biomedicine and Health, Chinese Academy of Sciences;<br>Vero E6 cells: ATCC, CRL-1586;<br>CHO: GenScript.
Authentication	Pseudovirus testing:<br>• 293T-ACE2 cells, not authenticated.<br>Live virus neutralization assay:<br>• Vero E6 cell line: not authenticated.<br>Antibody verification:<br>• 293T cells: authenticated by STR analysis.
Mycoplasma contamination	The cell lines were not contaminated by mycoplasma as determined by using the Lonza Mycoplasma Detection Kit.
Commonly misidentified lines<br>(See ICLAC register)	No commonly misidentified cell lines were used.

Flow Cytometry

Plots

Confirm that:

☒ The axis labels state the marker and fluorochrome used (e.g. CD4-FITC).

☒ The axis scales are clearly visible. Include numbers along axes only for bottom left plot of group (a 'group' is an analysis of identical markers).

☐ All plots are contour plots with outliers or pseudocolor plots.

☒ A numerical value for number of cells or percentage (with statistics) is provided.

Methodology

Sample preparation	PBMCs were isolated immediately from fresh blood by Ficoll (GE Healthcare) centrifugation. CD19+ B cells were enriched from pooled PBMCs using a CD19 MicroBeads kit (Miltenyi, Cat# 130-050-301). The enriched CD19+ B cells were then stained

with PE anti-human CD27 antibody (BD Biosciences, Cat# 566944), SARS-CoV-2 biotinylated RBD protein (His tagged) conjugated with FITC-streptavidin, and biotinylated S1 protein (His-tagged) conjugated with APC-streptavidin. CD19+CD27 +RBD+S1+ B cells were sorted with a BD AriaFusion flow cytometer.

Instrument	BD AriaFusion
Software	FlowJo v10
Cell population abundance	About 100000 events were recorded and a series of hierarchical gates were applied to isolate the target cells. 10000 cells were gating within lymphocytes population (enriched CD19+ cells). CD27+ cells (20%-40%) were gated within CD19+ cells population. 0.5%-3% SARS-CoV-2 specific cells were gated within CD27+ cells.
Gating strategy	Lymphocytes population was gated using FSC-A/SSC-A dot plot, then CD27+ cells were gated within CD27 PE/FAC-A under lymphocytes population (enriched CD19+ cells). SARS-CoV-2 specific cells were gated within RBD+S1+ under CD27+ cells population.

☒ Tick this box to confirm that a figure exemplifying the gating strategy is provided in the Supplementary Information.
